# Balloon Tamponade Utilization for Severe Esophagitis Causing Hemorrhagic Shock

**DOI:** 10.7759/cureus.17172

**Published:** 2021-08-14

**Authors:** Sarang Thaker, Gregory Pajot, Adam E Mikolajczyk, Anna M Lipowska

**Affiliations:** 1 Department of Medicine, Division of Gastroenterology and Hepatology, University of Illinois at Chicago, Chicago, USA

**Keywords:** esophagitis, non-variceal upper gastrointestinal bleeding, balloon tamponade, esophagogastroduodenoscopy, hemorrhagic shock

## Abstract

Esophagitis causing upper gastrointestinal bleeding (UGIB) is associated with significant morbidity. We present a case report of two patients with hemorrhagic shock secondary to esophagitis. Both patients underwent esophagogastroduodenoscopy demonstrating severe bleeding pan-esophagitis complicated by hemodynamic instability. Balloon tamponade for hemostasis was performed with resultant hemodynamic improvement. Severe UGIB secondary to esophagitis is difficult to control, with a high risk of complications and limited available endoscopic therapies in extensive mucosal injury. Treatments such as angiography are ineffective due to collateralization and surgery carries high morbidity and mortality. Balloon tamponade provides a rescue option for severe, refractory UGIB secondary to esophagitis.

## Introduction

After peptic ulcer disease and erosive gastritis and duodenitis, esophagitis is the most common cause of upper gastrointestinal bleeding (UGIB), and its incidence is increasing [1]. Current treatments include medical and endoscopic therapies, but guidelines are limited in patients with refractory bleeding causing hemodynamic instability. To date, literature is lacking on the use of balloon tamponade in non-variceal UGIB. We report a case series of patients with hemorrhagic shock secondary to esophagitis who required balloon tamponade for hemostasis.

This case report was accepted as an abstract in the International Society for Diseases of the Esophagus annual meeting, which was scheduled for August 19-21, 2020 in Toronto, Canada, but was postponed due to the coronavirus pandemic. The case report was not presented but instead published as an abstract in the Diseases of the Esophagus annual meeting supplement issue.

## Case presentation

Case 1

**Figure 1:**
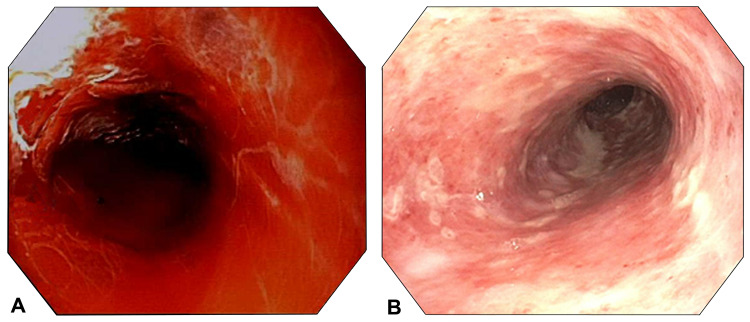
Case 1 endoscopy A: Case 1 index endoscopy demonstrating bleeding esophagitis. B: Case 1 post-balloon tamponade endoscopy demonstrating resolution of bleeding.

A 55-year-old male with a history of alcoholism, withdrawal seizures, alcoholic hepatitis, chronic pancreatitis, and Los Angeles (LA) grade D esophagitis was admitted to the intensive care unit for seizures, alcoholic hepatitis, and renal failure. The patient developed hemorrhagic nasogastric tube output with melena causing shock requiring massive transfusion protocol and vasopressors. Lab reports revealed anemia with hemoglobin 6.2 gm/dL from 9.6 gm/dL, thrombocytopenia to 74 thous/uL, and coagulopathy with an international normalized ratio (INR) of 1.6. Imaging demonstrated hepatomegaly with steatosis without sequelae of portal hypertension. Proton pump inhibitor (PPI), octreotide, and blood products were infused. Esophagogastroduodenoscopy (EGD) demonstrated fresh blood throughout the esophagus, no evidence of esophageal varices or ulcers, and bleeding LA grade D esophagitis at 25-40 cm from the incisors (Figure 1A), extending to the gastroesophageal junction. Given the extent and severity of mucosal injury with no localized source, presence of large blood clots, and limited visualization, no endoscopic therapies were performed. The hemostatic powder was also not available at the time of this case. Computed tomography angiography (CTA) revealed active distal esophageal bleeding with feeding vessels from dilated esophageal branches of the left gastric artery. The patient subsequently underwent embolization of a left gastric artery branch with a resolution of bleeding from that distribution; however, there was mildly persistent extravasation from collateral vessels on post-intervention angiography. Given the emergent nature of the case and lack of availability, a through-the-scope esophageal stent was not offered.

After the patient was deemed a high-risk surgical candidate and repeat EGD redemonstrated diffuse active esophageal bleeding, a Minnesota tube was placed. Given continued bleeding after gastric balloon inflation to 400 mL, the esophageal balloon was inflated to 30 mmHg. Aspiration confirmed hemostasis and the esophageal balloon was periodically deflated to minimize the risk of necrosis and perforation. Transfusion and vasopressor requirement resolved. Both balloons were deflated after six hours. EGD performed the following day showed non-bleeding severe pan-esophagitis (Figure 1B). The bleeding did not recur, and the patient was discharged days later.

Case 2

A 44-year-old male with decompensated alcoholic cirrhosis, pulmonary hypertension, and mixed connective tissue disease was admitted with renal failure and pneumonia. He developed hematemesis and melena with an acute decrease in hemoglobin from 7.6 gm/dL to 3.9 gm/dL, thrombocytopenia to 25 thous/uL, coagulopathy with INR 2.0, and shock. PPI, octreotide, and blood products were administered.

**Figure 2:**
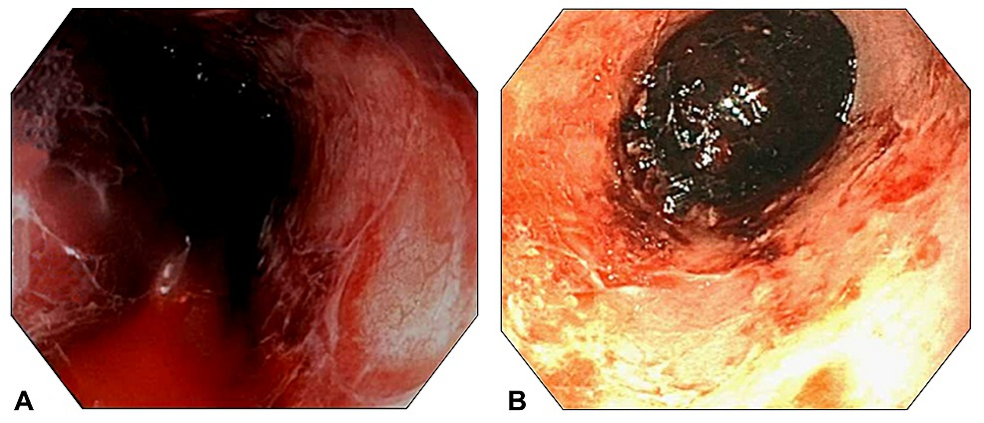
Case 2 endoscopy A: Case 2 index endoscopy demonstrating bleeding esophagitis. B: Case 2 post-balloon tamponade endoscopy demonstrating resolution of bleeding.

EGD demonstrated extensive bleeding LA grade D esophagitis and a large, partially circumferential, immobile thrombus in the middle and lower thirds of the esophagus (Figure 2A). The esophageal mucosa was diffusely friable with bleeding from numerous sites without a discrete culprit lesion such as an ulcer. More rapid bleeding was seen emanating from within the thrombus but attempts to dislodge the thrombus were unsuccessful. The hemostatic powder was then applied several times but this was still unsuccessful in achieving hemostasis. The application was technically challenging due to the rate of bleeding in the confined space of the esophagus repeatedly leading to occlusion of the catheter tip. Additionally, the large thrombus shielded the underlying mucosa from the delivery of the powder. Therefore, a Minnesota tube was inserted with the gastric balloon inflated to 400 mL and set to traction for 12 hours. Once again, given the emergent nature of the case and lack of availability, a through-the-scope esophageal stent was not offered. Following placement, hemostasis was achieved, with stabilization in hemodynamics and transfusion requirements. Imaging such as CTA was not obtained due to clinical improvement. Post-deflation EGD demonstrated LA grade D esophagitis 20-33 cm from the incisors (Figure 2B) with the gastroesophageal junction noted at 38 cm. The hemostatic powder was reapplied for minor oozing. Several days later, the patient developed septic shock and expired.

## Discussion

Esophagitis is the etiology of 4-12% of UGIB events and carries substantial morbidity [2]. Guntipalli et al. determined that hematemesis was more common in esophagitis compared to other etiologies of UGIB [3]. Furthermore, as severity worsened, so did the frequency of hematemesis with concurrent melena as in our cases.

With regards to liver disease, Guntipalli et al. noted that patients with cirrhosis and esophagitis received more blood products, had higher rebleeding rates, and had the highest mortality rate of all groups examined [3]. The reason for these findings is not exactly known. Coagulopathy due to liver disease likely contributes to bleeding severity as observed in the cases above. Moreover, portal hypertension also likely contributes and may worsen outcomes [4]. Portal hypertension was definitively observed in the patient who died whereas the presence of portal hypertension is unclear in the patient who survived.

Regarding esophagitis risk factors, Zimmerman et al. concluded that age is an independent risk factor [5]. Guntipalli et al. noted that gastroesophageal reflux disease was more common in patients with esophagitis [3]. Multivariate analysis in patients with esophagitis revealed that increased age, psychotropic medications, and higher LA grades were significantly associated with complications [6].

Guidelines on UGIB management recommend resuscitation, acid suppression with PPI, and endoscopic evaluation within 24 hours [7]. However, these modalities are unlikely to be effective when there is extensive bleeding. In patients with refractory bleeding who fail endoscopic management, guidelines recommend either surgical or interventional radiology consultation. Emergent surgery is associated with elevated morbidity and mortality. Czymek et al. found a mortality rate of 34.1% associated with surgical interventions [8]. Loffroy et al. found a 67% success rate of transcatheter arterial embolization (TAE) for UGIB [9]. Risk factors for failure include coagulopathy, collateralization, delay to angiography, and a greater number of transfusions [9]. The esophagus is highly vascularized with a rich arterial supply from branches of the inferior thyroid artery, aortic esophageal arteries, bronchial arteries, and left gastric artery [10]. Vogten et al. report that this complex and redundant arterial network of the esophagus creates a technical challenge for TAE as bleeding may be of multi-vessel origin [11].

Balloon tamponade is a rescue method for variceal bleeding and its utilization for non-variceal UGIB is not well described. Chen et al. reported the use of balloon tamponade in a patient with ulcerative esophagitis that failed endoscopic treatments [12]. Gastric balloon inflation resulted in clinical improvement, similar to the cases above. Hemostasis is achieved via mechanical compression of the vasculature; however, post-deflation rebleeding is common [13]. In patients with upper and mid esophageal bleeding, inflation of the gastric balloon may not be effective, and consideration of esophageal balloon inflation is necessary. In case 1, the patient was noted to have ongoing extravasation from collaterals proximal to the gastroesophageal junction after left gastric arterial embolization, which explains the lack of hemostasis with gastric balloon inflation alone. A meta-analysis reported a 35% failure rate and a complication rate of nearly 10% [14]. Complications of balloon tamponade include airway compression, aspiration, tissue necrosis, and esophageal perforation due to intra-esophageal gastric balloon inflation or prolonged esophageal balloon inflation. Recommendations are to limit esophageal balloon inflation pressure to 35-45 mmHg, use the lowest pressure necessary, inflate for a maximum of 24 hours, and periodically deflate with lavage to assess for hemostasis [15].

The hemostatic powder is an option for refractory gastrointestinal bleeding secondary to a wide range of etiologies including esophagitis, peptic ulcer disease, and malignancy. The hemostatic powder is activated by fluids and results in clot formation over bleeding vessels within moments. A recent meta-analysis by de Rezende et al. reported a rate of hemostasis of 90.7%; however, the rebleeding rate within 72 hours was elevated at 26.2% [16]. Unfortunately, this modality is expensive and may not be widely available. The application of hemostatic powder requires active bleeding, and the delivery system must remain dry to avoid catheter clogging and malfunction. Once applied, the endoscope should not be near the targeted area to avoid inadvertent clearance. In case 2 above, hemostatic powder was ineffective due to contact of blood with the catheter tip and bleeding originating from mucosa that was obscured by the thrombus.

The use of self-expanding covered esophageal stents has been shown to be effective in controlling bleeding related to varices, but not studied in other etiologies of UGIB. Escorsell et al. conducted a multicenter randomized control trial comparing stents to balloon tamponade in refractory esophageal variceal bleeding. Stents were found to be more effective in achieving hemostasis and were associated with fewer adverse events [17]. Self-expanding covered stents work in a similar fashion as balloon tamponade by applying pressure to the mucosa and compressing vessels. Jain et al. reported that esophageal stents may also be used to control bleeding secondary to post-banding esophageal ulcers [18]. However, safe placement of a stent requires additional training and experienced staff that may not be available in emergencies, especially if fluoroscopy is necessary when through-the-scope stents are not offered. One potential advantage is the ability to stent bleeding areas in the mid and upper esophagus, which may be hard to control with balloon tamponade; however, stent migration can occur which would render the stent ineffective. Alternatively, stenting is limited to the length of the device and may not achieve hemostasis in diffuse bleeding. Importantly, there are no studies or case reports on the use of esophageal stents for UGIB due to esophagitis.

## Conclusions

In the evaluation of UGIB, esophagitis and its limited treatments must be considered. Endoscopy can provide diagnostic information, but diffuse esophageal bleeding is often not amenable to endoscopic therapy. The abundant vascular supply of the esophagus limits radiology interventions and surgery is associated with elevated risks. The presence of liver disease with esophagitis warrants attention as this population is at elevated risk. In these two cases of extensive esophagitis causing hemorrhagic shock, balloon tamponade was essential to achieving hemostasis and clinical stability as other modalities were either ineffective or unavailable. Balloon tamponade should be a consideration in similar cases with refractory bleeding. Literature on hemorrhage from esophagitis in patients with and without liver disease is deficient and should be a focus in future research.
